# Multilocular Ovarian Cyst

**Published:** 1872-08

**Authors:** John C. Gooding

**Affiliations:** Cheltenham


### Multilocular Ovarian Cyst never tapped: Ovariotomy; Recovery.

———

By Dr. John C. Gooding, Cheltenham.

[The patient was sixty years of age and unmarried. She had always had excellent health. The symptoms of the disease dated back eight years. Just before the operation was performed the abdomen was entirely filled by a fluctuating tumor, extending above the ensiform cartilage; the uterus was mobile, and in its normal condition; the tumor could not be felt by the vagina.]

June 15th, 1871. The patient is in excellent health, and was out even up to yesterday making little purchases in preparation for her seclusion. She is hopeful but resigned. The bowels acted during the night, and the bladder was emptied before the operation. Beef-tea was given three hours, and brandy-and-water immediately before the administration of the chloroform, which was kindly undertaken by Mr. C. J. Bennet. The patient, warmly clad, was conveniently placed on a table in a room the temperature of which was 70° F. An incision, three inches long, was carried midway between the umbilicus and symphysis pubis, through the skin, fasciæ, and rectus, down to the peritoneum, which was then divided on a director. The hand, introduced between the tumor and abdominal walls, found no adhesions, not even in the left iliac region, where I expected there might have been some resulting from the recent inflammatory attack. The cyst was punctured, and withdrawn until it resisted gentle traction. To introduce the hand to determine the detaining cause it was necessary to extend the incision by an inch, as the partially extruded cyst occupied a portion of the wound. I found, high up in the epigastrium another cyst, the contents of which were evacuated through the first, and then the whole growth was easily withdrawn. The pedicle, very short and thin and two inches broad, was transfixed through a translucent part and tied in two portions with stout silk, severed, and replaced in the pelvis. The other ovary was healthy. The insignificant oozing from the divided rectus was sponged away; no fluid having escaped into the abdomen (thanks to the efficient assistance of Messrs. J. Humphreys and C. J. Newton). The wound was closed by five silver sutures traversing the peri onium at least half an inch from its cut edges, and two or three superficial wire sutures and broad strips of plaster. A large pad of cotton wool, retained by other strips of plaster, filled and supported the concave abdomen and completed the dressing. The patient was replaced in bed; pulse 85, good.

The tumor was made up of two large cysts, which between them held twenty-five pints of thick dark fluid; a third cyst, about the size of an orange, containing glairy white fluid; and numerous small cysts, embedded in and projected from the interior of the walls of the larger cysts, and crowding round the pedicle. When the cysts were all emptied, the solid portion weighed 2 lb. 10 oz.

Four hours after the operation the patient was comfortable; had felt slight nausea, which was completely allayed by ice; the pulse was 96; skin warm and perspiring. Eight ounces of urine drawn off. One tablespoonful of cold milk was given, and ice only ordered for the next few hours. At midnight—eight hours after the operation—I found that the patient had slept soundly for an hour; her aspect was good; pulse 90; skin warm and moist. Six ounces of urine drawn off. On the following day I fonnd that the patient had taken thirty minims of nepenthe to relieve slight pain occasioned by the vermicular action of the intestines; she had slept well, and was comfortable. A teacupful of milk, and half that quantity of beef tea, had been taken during the past twenty-four hours. On the third day flatus passed, the patient continuing her favourable progress. On the seventh day all the sutures were removed, as the wound was found to have healed throughout its entire length; but at the upper angle, from the want of another superficial suture, one lip was more elevated than the other, exposing half an inch of raw surface. A piece of lint soaked in carbolic acid lotion, and long strips of plaster, embracing the lips, were applied; the wool and plaster as before to support the abdomen, which continued undistended. The use of the catheter was continued up to the tenth day, from the patient's inability voluntarily to evacuate the bladder. The bowels were relieved by castor oil on the ninth day. On the twelfth day a tonic mixture was ordered, to stimulate her appetite; on the fourteenth she was allowed to sit up in bed; and on the twenty-first, the wound having for some days past completely healed, she took an airing in a wheel chair. A few days after she went into the country; and on her return recently came to see me, and was looking exceedingly well.

To tie and return the pedicle certainly seems the next best mode of dealing with it, when, owing to its shortness, the clamp cannot be used. The portion of the pedicle on the distal side of the ligature—the stump,—surrounded as it is by *warm* tissues, no doubt ritains its vitality long enough for it to become attached by lymph—rapidly effused and organized as we know this to be—to adjacent parts. And in the same way that a completely detached portion of lip, if quickly readjusted, *and its warmth be maintained,* will soon become part of the body again, so does the stump become vivified by blood conveyed to it through the newly formed vessels rapidly developed in the effused lymph. The ligature, when tightened, buries itself too, and brings into apposition the peritoneal covering of the pedicle on either side of it, and between these adhesion probably soon takes place. The *material* of the lagature would scarcely seem, theoretically, to affect the result, for by the complete closure of the abdominal wound air is excluded and decomposition prevented; there being then no putrefying fluid for the ligature to absorb, hemp and silk would be on an equality with metal. The results of cases by those who have had many opportunities of treating the pedicle as in this case seem, practically, to favour this view.—*Lancet, Dec.* 2, 1871. *Braithwaite's Retrospect.*

